# IMGT/Collier-de-Perles: a two-dimensional visualization tool for amino acid domain sequences

**DOI:** 10.1186/1742-4682-10-14

**Published:** 2013-02-21

**Authors:** Dimitrios Vlachakis, Christos Feidakis, Vasileios Megalooikonomou, Sophia Kossida

**Affiliations:** 1 Bioinformatics & Medical Informatics Team, Biomedical Research Foundation, Academy of Athens, Soranou Efessiou 4, Athens, 11527, Greece; 2 Computer Engineering and Informatics Department, School of Engineering University of Patras, Patras, 26500, Greece

**Keywords:** IMGT/Collier-de-Perles, IMGT Collier de Perles, Graphical representation, Protein domains

## Abstract

IMGT/Collier-de-Perles is a tool that allows the user to analyze and draw two-dimensional graphical representations (or IMGT Collier de Perles) of protein domains (e.g., hydropathy plots). The IMGT/Collier-de-Perles specializes in the area of immunoglobulins (IG) or antibodies, T cell receptors (TR) and major histocompatibility (MH) of human and other vertebrate species as well as other proteins of the immunoglobulin superfamily (IgSF) and of the major histocompatibility superfamily (MhSF) and related proteins of the immune system of vertebrates and invertebrates.

## Introduction

Amino acids can be defined and classified in a number of ways, depending on the perspective they are being examined from each time. Thereby, they can be categorized according to the functional groups of their side chains, which determine their physicochemical characteristics [1].

Taking into account the importance of proteins, made of amino acids, as a structural component of all living organisms, the significance of a method or tool that could seamlessly manipulate this data would be extremely practical. Indeed, scientists have been using computational tools that enable them to compare and examine amino acid sequences in a number of ways.

Among the different classes of amino acid properties, hydrophobicity determines how strongly an amino acid is attracted to or repelled by water. A series of different hydrophobicity scales have been developed [2-8]. The higher the index value is in a scale, the more hydrophobic is the amino acid. Differences between the scales mainly depend on the method or on the algorithm used to measure or to define hydrophobicity [6,9-12]. Hydrophobicity scales are commonly used to predict the leader region (or signal peptides) or the transmembrane region of proteins. When measuring sequential amino acids of a protein, fluctuations in value indicate protein hydrophobic regions potentially located inside the membrane lipid layer [13] or contributing to the hydrophobic core of a protein [2]. Hydropathy and other amino acid properties are keys for a better understanding of protein interactions and domain structures.

## The IMGT/Collier-de-Perles tool

The IMGT/Collier-de-Perles [14] tool was created by LIGM (Université Montpellier 2, CNRS) and is part of IMGT®, the international ImMunoGeneTics information system® [15,16] (IMGT®, http://www.imgt.org), which is acknowledged as the global reference in immunogenetics and immunoinformatics.

**Figure 1:**
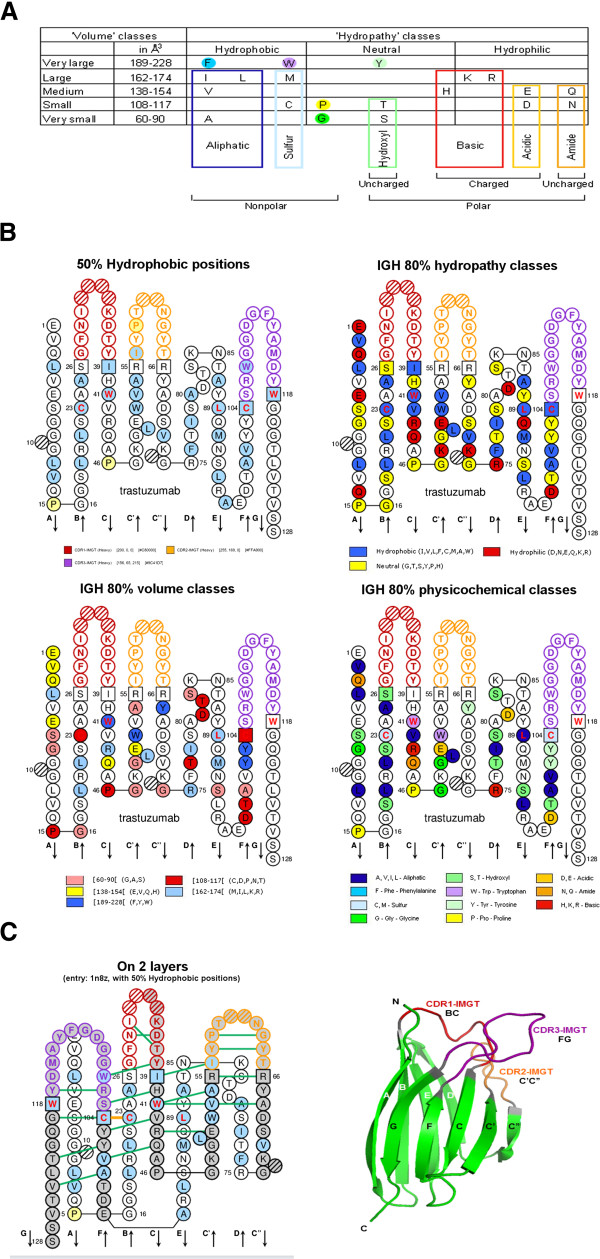
**Amino acid properties and the IMGT Collier de Perles 2D hydropathy plot.** (**A**): The 11 IMGT physicochemical classes of the 20 common amino acids [1]. (**B**): IMGT Colliers de Perles of a V domain of an IG or antibody. IMGT Collier de Perles on one layer, obtained by the IMGT/Collier-de-Perles online tool [14] at the IMGT Home page, http://www.imgt.org. Four options are shown for the V domain of the heavy domain (VH) of trastuzumab, a therapeutic antibody: ‘50% hydrophobic positions’ (with that option, an amino acid at a given position is colored in blue if an hydrophobic amino acid is found at that position in 50% or more of the sequences used for establishing the statistical profile [17], ‘IGH 80% hydropathy classes’, ‘IGH 80% volume classes’, ‘IGH 80% physicochemical classes’. The CDR-IMGT lengths are [8.8.13]. The five conserved amino acid positions of a V domain 23, 41, 89, 104 and 118 (1st-CYS C23, CONSERVED-TRP W41, hydrophobic 89, 2nd-CYS C104, J-PHE or J-TRP 118, here W118) appear with red and bold letters. The six anchors (positions 26 and 39, 55 and 66, 104 and 118) which belong to the framework regions (FR-IMGT) and support the complementarity determining regions (CDR-IMGT) are in squares. (**C**): IMGT Collier de Perles on 2 layers and with hydrogen bonds (green lines) of the trastuzumab VH , provided by IMGT/3Dstructure-DB [26,34] (PDB code 1n8z). These hydrogen bonds are from the experimental 3D structures and are displayed by the IMGT/Collier-de-Perles tool integrated in IMGT/3Dstructure-DB [26,34]. The 3D structure representation of a V domain with its strands and loops, based on the IMGT Collier de Perles delimitations [21,22,25], is also shown.

IMGT/Collier-de-Perles can provide upon selection three types of displays: the hydropathy plot with 3 classes (hydrophobic, neutral, hydrophilic), the volume plot with 5 classes, and the physicochemical plot, which is the most informative one, with eleven IMGT physicochemical classes (which were defined taking into account hydropathy, volume and chemical characteristics properties) [1,17] (Figure 1A). Eleven IMGT physicochemical classes of the 20 common amino acids have been defined by the physicochemical properties of their side chains [17]. These standardized classes are used in the IMGT/Collier-de-Perles tool.

IMGT Colliers de Perles can currently be drawn for three domain types: variable (V) domain and constant (C) domain of immunoglobulins (IG) or antibodies and T cell receptors (TR) and immunoglobulin superfamilies (IgSF) proteins other than IG and TR, and groove domain (G) of the major histocompatibility (MH) and MH superfamily (MhSF) other than MH [18-21]. In order for an IMGT Collier de Perles to be created, each sequence has to be gapped according to the IMGT unique numbering [22-25], using IMGT/DomainGapAlign [26-28].

IMGT/DomainGapAlign allows the creation of gaps in the user’s V, C or G domain amino acid sequence, by aligning the user sequence to the corresponding IMGT domain reference directory and identifies, for the IG or TR V domain, the closest germline V-REGION and J-REGION, and for all other cases (V domain of IgSF other than IG or TR, C domain and G domain) the closest V, C or G domain of the reference gene and/or allele, respectively, and finally obtains the IMGT Collier de Perles. Amino acids which differ from the closest reference sequence are highlighted in the IMGT Collier de Perles (pink border, online) and the IMGT amino acid change characteristics detailed in accompanying tables [26-28].

The resulting IMGT Colliers de Perles (Figure 1B) help us determine which amino acids are important for the 3D structural configuration and, for the IG and TR V domain, delineate the standardized framework regions (FR-IMGT) (formed by the nine antiparallel beta strands) and complementarity determining regions (CDR-IMGT) (formed by the three loops binding the antigen). The length of the strands, loops and turns in IMGT Colliers de Perles provide critical information in the characterization of each V, C or G domain [21,25].

## How can we use IMGT Colliers de Perles?

The first among numerous features of the IMGT Collier de Perles is based on the way that domains of the antibody or T cell receptor are characterized. Each domain is described by the length of its loops, strands and turns, and helix (for G domain) [21,25]. This way, the usual but confusing distinction made in the literature and generalist databases, between C1, C2 and I1 sets is often inappropriate upon the absence of structural data and can be ignored [23].

Unlike generalist databases such as UniProt/Swiss-Prot, IMGT standardization defines the different domains by comparing amino acid or cDNA sequences with genomic sequences, therefore identifying the splicing sites and giving a more accurate delimitation of the existing domains [23].

Another strong feature of the IMGT Collier de Perles is that it produces a standardized graphical representation and allows to visualize and to localize the differences between domains of proteins whatever the species even when 3D data are unavailable [29]. This can be a great tool for molecular engineers. Antibody humanization, particularly, is greatly benefited by an interface that seamlessly displays and compares FR-IMGT and CDR-IMGT among several species [30-33].

IMGT Collier de Perles can also be used to compare a given amino acid sequence against an IMGT reference sequence, in order to facilitate the identification of potential immunogenic residues at certain positions of humanized antibodies or to assess the immunogenicity of therapeutic antibodies (Figure 1B). The reference sequence is in essence a statistical profile created for the human IG heavy, kappa and lambda expressed variable domains sets and is based, as the physicochemical plots, on the description of the 11 IMGT amino acid physicochemical classes (that include hydropathy, volume and chemical characteristics of the 20 common amino acids) [17].

Besides being a 2D visualization tool, IMGT/Collier-de-Perles can also take advantage of 3D structures when those are available [26,34], by displaying IMGT Colliers de Perles on two layers with hydrogen bonds between amino acids of V or C domains of the antibody (Figure 1C). In Figure 1C, the FR-IMGT is made up of 9 strands (arrows) and turns is in green and the 3 CDR-IMGT are in red, orange and purple, respectively (http://www.imgt.org, The IMGT Biotechnology page, Antibody engineering, FR-IMGT and CDR-IMGT).

Additional information such as atom contact types and categories can be provided for each amino acid separately, by clicking on each amino acid in the IMGT Collier de Perles.

## Conclusion

Based on the IMGT unique numbering concept generated from the NUMEROTATION axiom of IMGT-ONTOLOGY [16], the IMGT/Collier-de-Perles tool is a very friendly tool which allows users to create visual representation of their own amino acid sequences for V, C and G domains [21,25]. The tool can be used on its own or as an output functionality of IMGT/DomainGapAlign [26-28]. It has also been integrated in IMGT/3Dstructure-DB (http://www.imgt.org), the IMGT three-dimensional (3D) structure database [26,34]. Thus the users can compare their own IMGT Colliers de Perles with those provided in the database for analysis of interactions, e.g., those of the V domains of IG and TR in complex with their antigen (query on IG/Ag and TR/pMH) or those of the C or G domains of the FcR in complex with the IG Fc. IMGT Colliers de Perles provide a great help for understanding relations between sequences and structures in the design of therapeutic monoclonal antibodies (antibody engineering and humanization) [30,31,33] with their functional properties (specifity, affinity, immunogenicity, allotype expression [32], etc.), and more generally for characterizing the V, C and G domains of the proteins belonging to the IgSF and MhSF superfamilies of all vertebrates and invertebrates.

## Competing interests

The authors declare that they have no competing interests.

## Authors’ contributions

DV and SK conceived, coordinated, supervised and designed the study. DV, CF, VM and SK carried out the study and drafted the manuscript. All authors read and approved the final manuscript.
